# Chlorambucil plus Rituximab as Front-Line Therapy in Elderly/Unfit Patients Affected by B-Cell Chronic Lymphocytic Leukemia: Results of a Single-Centre Experience

**DOI:** 10.4084/MJHID.2013.031

**Published:** 2013-05-02

**Authors:** Luca Laurenti, Barbara Vannata, Idanna Innocenti, Francesco Autore, Francesco Santini, Nicola Piccirillo, Tommaso Za, Silvia Bellesi, Sara Marietti, Simona Sica, Dimitar G. Efremov, Giuseppe Leone

**Affiliations:** 1 Department of Hematology, Catholic University of Rome, “A. Gemelli” Hospital, Largo A. Gemelli 8, Rome, Italy.; 2 Department of Molecular Hematology, International Centre for Genetic Engineering & Biotechnology, Campus A. Buzzati-Traverso, Rome, Italy.

## Abstract

The current standard first line therapy for fit patients with B-CLL/SLL is based on combination of fludarabine-cyclophosphamide and rituximab. However, elderly patients or patients with comorbidities poorly tolerate purine analogue-based chemotherapy and they are often treated with Chlorambucil (Chl) only. However, complete response (CR) and overall response (OR) rates with Chl are relatively low. We now investigated whether the addition of Rituximab to Chl will improve the efficacy without impairing the tolerability in elderly and unfit patients. We included in our study 27 elderly or unfit patients that had not received prior therapy. All patients were treated with Chl (1mg/Kg per 28-day cycle for 8 cycles) plus Rituximab (375 mg/m^2^ for the first course and 500 mg/m^2^ for subsequent cycles until the 6^th^ cycle). We obtained an OR rate of 74%. The most frequent adverse effect was grade 3–4 neutropenia, which occurred in 18.5% of the patients. Infections or grade 3–4 extra-hematological side effects were not recorded. None of the patients required reduction of dose, delay of therapy or hospitalization. Overall, these data suggest that Chl-R is an effective and well tolerated regimen in elderly/unfit patients with CLL.

## Introduction

Chronic lymphocytic leukemia (CLL) is the most prevalent adult leukemia in western countries, with an incidence reaching about 13 per 100.000 at 65 years, which is the median age of onset of the disease.^1^

Chlorambucil, an alkylating agent, was the standard first line treatment for B-CLL/SLL before the development of the purine analogues. This changed from 2000, when phase III trials showed an improved ORR and a prolongation of PFS with fludarabine.^2^ Later, a further improvement was obtained by combining Fludarabine with Cyclophosphamide (FC).^3^ The subsequent addition of the monoclonal anti-CD20 antibody Rituximab to the Fludarabine/Cyclophosphamide combination (FCR) resulted in an even more effective regimen that has become the standard first line therapy for CLL.^4^ However, the FCR regimen can result in significant myelosuppression and a high rate of early and late infections, especially in elderly patients with CLL, suggesting that it may be too toxic and therefore unsuitable for this large subpopulation of patients.^5^,^6^

Chl as a single agent is well tolerated among elderly patients with CLL. In the LRF CLL4 trial it compared favourably with fludarabine with respect to myelotoxicity, neutropenia and fever and showed similar progression-free survival.^7^ In addition, data from the CLL 5 phase III trial of the German CLL study group (GCLLSG) comparing fludarabine vs. Chlorambucil in patients older than 65 years displayed no differences in OS and PFS between fludarabine and Chl, despite a greater percentage of CR and ORR with fludarabine.^8^ Notably, the fludarabine group demonstrated a shorter median survival time and higher rate of toxicity, indicating that there is no major clinical benefit of using fludarabine over chlorambucil in elderly CLL patients.

Since Rituximab is well tolerated in elderly CLL patients and results in improved responses and outcome when combined with Fludarabine or FC, an obvious combination therapy in the setting of elderly/unfit patients would be immunotherapy with Rituximab and Chl. We therefore assessed the efficacy and safety of this combination in a series of previously untreated, elderly or unfit B-CLL patients.

## Materials and Methods

### Patients and Treatment

Previously untreated elderly patients ≥ 60 years old or unfit pts > 18 years old with B-CLL or Small Lymphocytic lymphoma (SLL), according to the WHO classification 2008, were included in the study. Patients were considered unfit if CIRS score was > 6.^9^–^11^ Patients included in the study were required to have ECOG ≤ 2 to receive the planned treatment as outpatients. All patients provided written informed consent. This study was approved by an internal Ethics Committee.

The planned treatment schedule consisted of Chlorambucil 1 mg/Kg for each cycle every 28 days p.o. administered at a standard daily dose of 10 mg starting from day 1 and repeated for 8 cycles. Rituximab was added to Chl from the 3^rd^ cycle onwards and was administered on day 1 of each cycle at a dose of 375 mg/m^2^ during the first administration and at 500 mg/m^2^ for the subsequent 5 cycles. In patients with high lymphocyte counts the first dose of Rituximab was split into three days from day 1 to day 3 to prevent Tumour Lysis Syndrome. Approximately 30 minutes prior to Rituximab infusion all patients received pre-medication with oral acetaminophen (650–1000 mg) and an antihistaminic, while patients with high lymphocyte counts also received prednisolone at a dose of 1mg/kg. Reduction of 25% of the dose and a minimum of 6 cycles of Chl and 4 cycles of RTX were allowed. Prophylaxis against *Pneumocystis carinii* was provided by administration of trimetoprim-sulfametoxazole 960 mg bid for two consecutive days every week. Patients with active HBV or HCV infection were excluded from the study. Prophylaxis with lamivudine at the dose of 100 mg/day was administered to patients with serum positivity for antibodies against HBcAg and/or HBeAg of Hepatitis B virus (HBV) until 6 months after the last cycle. G-CSF was administered for Grade IV neutropenia. Patients who progressed or developed autoimmune hemolytic anemia or thrombocytopenia during treatment, or required a dose reduction ≥ 25% were excluded from the study. The response evaluation at the end of treatment consisted of peripheral blood counts, physical examination, bone marrow aspiration with cytofluorimetric analysis and ultrasound or CT scan.

### Study End-Points

The primary objectives of this study were to assess the efficacy and safety of Chlorambucil plus Rituximab as front-line therapy in elderly and/or unfit patients with progressive CLL. As secondary endpoints we studied the influence of biological parameters, such as IGHV gene mutation status, expression of Zap-70 and CD38 and the presence of cytogenetic abnormalities or bulky disease, on the overall response rate (ORR), progression free survival (PFS) and time to retreatment (TTR). Bulky disease was defined as the presence of lymph-nodes more than 5 cm in longest diameter by CT and/or ultrasound examination or the presence of massive splenomegaly (6 cm below the left costal margin). High risk (HR) cytogenetic abnormalities were considered the presence of del(11q) and del(17p), while the absence of cytogenetic abnormalities and the presence of del(13) or trisomy 12 were considered as standard-risk (SR). Standard risk group included both low and intermediate cytogenetic risk according to Döhner’s hierarchical model.^12^ Response assessment was evaluated according to the updated National Cancer Institute-Working Group guidelines.^13^ Adverse effects were graded according to the NCI CTCAE v 4.0 scale (http://ctep.cancer.gov/reporting/ctc.html).

### Biological Studies

Fluorescent in situ hybridization (FISH), IGHV gene mutation analysis and flow-cytometry for Zap-70 and CD38 were performed using standard methodology, as described elsewhere.^12^

### Statistical Analysis

The overall survival (OS) time was calculated from the first day of treatment until death from any cause. The progression-free survival (PFS) was defined as the time interval from the first day of treatment until disease progression or death and time to retreatment (TTR) time was defined as the time interval from the first day of treatment until initiation of a new treatment or death. The survival curves were generated using the Kaplan–Meier method. The log-rank test was performed for survival curves comparison. The chi-square test was used to analyse the categorical variables. Statistical analysis was performed using the Statistical Package for Social Sciences (SPSS), release 16.0 for Windows. A p values less than 0.05 were considered significant.

## Results

### Patients Characteristics

Starting from May 2008 until May 2012, 27 patients with previously untreated progressive B-CLL or SLL were enrolled in the study. Eighteen patients were male and 9 were female, with a median age at the time of treatment of 72 years (range 58 to 85 years). Nine patients (33%) were unfit (CIRS>6) with a median age of 72 years (range 58 to 79 years). The other 18 elderly patients had a median CIRS of 3 and a median age of 71 years (range, 60–85 years). Six patients were in stage A/I, 12 were in stage B/II and 9 in stage C/IV. Eleven patients had an ECOG score of 0, 15 patients had an ECOG score of 1, while one patient had an ECOG score of 2. Median lymphocyte count at study entry was 50.000/mmc (range 4620 to 180.000). FISH analysis was done in all patients: 70% showed karyotype abnormalities. Standard-risk FISH karyotype was detected in 24/27 pts: 14 of them had del(13q14), 1 had trisomy 12, 1 trisomy 12 and del(13q14), and 8 had a normal karyotype. Three patients showed a high risk karyotype: all of them had del(11q). None of the patients had a 17p deletion. Zap-70 data were available for 26/27 pts, 8 of them (29.6%) were Zap-70 positive. CD38 was positive in 9/27 pts (33%). Twenty-two pts were studied for IGHV mutation status (82%), 14 had unmutated while 8 had mutated IGHV genes. Five patients showed bulky disease (Table 1).

### Responses

Twenty-four patients completed the planned treatment. Three pts, UPN7 UPN12 and UPN22, discontinued the treatment protocol because of the appearance of Autoimmune Hemolytic Anemia (AIHA) after the 2^nd^ cycle of Chl (UPN12) or because of disease progression after the 5^th^ and the 3^th^ cycle of Chl-Rituximab, respectively. The median number of Chl and Rituximab cycles administered in the 24 patients that completed the treatment protocol was 8 (range 6 to 8 cycles) and 6 (range 4 to 6 cycles) respectively. The median total dose of Chl administered during the treatment was 595 mg per patient (median dose 85 mg each cycle). The fixed daily dose of Chl was chosen to facilitate better compliance to the planned schedule by the patients. Four patients received a fractionated dose of Rituximab because of a high lymphocyte count. The median dose of Rituximab administered was 3500 mg per patient (median dose 620 mg each cycle).

On an intention to treat basis the OR rate was 74%. Seven pts (26%) obtained a complete response, 13 pts (48%) obtained a partial response. No statistically significant differences were noticed in terms of OR rate for age above or below 70 years, fitness status, bulky disease, cytogenetic risk abnormalities or IGHV, ZAP-70 and CD38 categories (Table 2).

### PFS, TTR, OS

**Figure 1:**
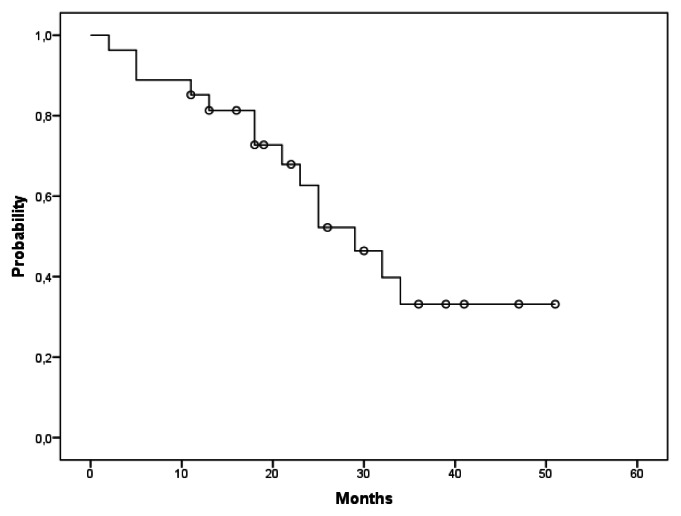
Kaplan-Meier curve of PFS. The Figure 1 shows the probability of survival free from progression by time (months) in the study population.

On an intention to treat basis the median PFS was reached at a median time of 29 months (Figure 1). Among fourteen pts who experienced progression after treatment, the median time was 19.5 months (range 2–36).

**Figure 2:**
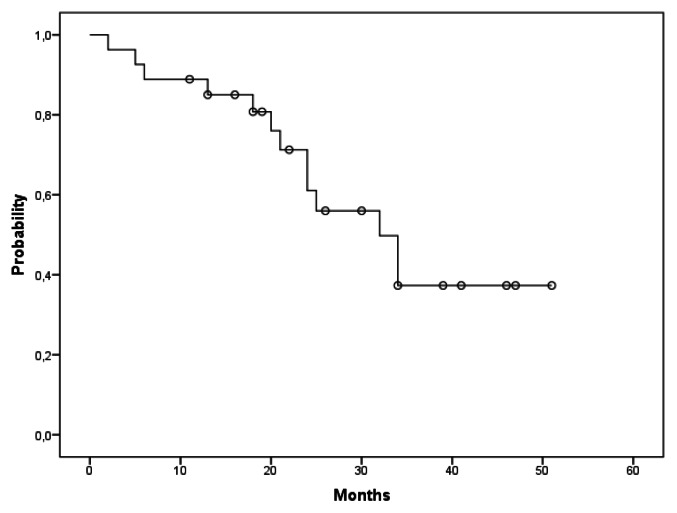
Kaplan-Meier curve of TTR. The Figure 2 shows the probability of re-treatment by time (months) in the study population

TTR was reached at a median time of 32 months (Figure 2). Thirteen pts received a second line of treatment at a median time of 22.5 months (range 2–34 months). At present, six patients died, the median follow-up is 30 months with an OS rate of 78%. No statistically significant differences were noticed for the examined risk categories with respect to PFS, TTR and OS. Although the difference was not statistically significant (p=0.064), the ‘high risk’ cytogenetic group showed more rapid disease progression with respect to the ‘standard risk’ cytogenetic group (18 vs 32 months respectively). (Table 2).

### Hematological and Extra-Hematological Toxicity

Four patients (15%) developed grade IV neutropenia, requiring administration of G-CSF and one patient showed grade III neutropenia (Table 3). One patient discontinued treatment because of AIHA. None of the patients developed autoimmune thrombocytopenia.

Regarding extra-hematological toxicity, a grade 2 infusion related reaction characterized by skin rash was recorded in 4 patients (15%) after the first and second administration of RTX and was treated with intravenous steroids. Two patients experienced nausea at the end of Chl administration and 1 patient experienced vomiting during the first and second cycle of therapy that was promptly resolved with ondansetron. None of the patients reported diarrhea. One patient experienced fever of unknown origin that was resolved with empiric antibiotic therapy and did not require hospitalization. None of the patients that completed the treatment protocol required hospitalization during the planned therapy. The patient who discontinued the treatment protocol because of AIHA was successfully treated with cyclophosphamide and rituximab. He also had CMV reactivation and was given therapy with valganciclovir until the CMV viremia became negative. None of the patients discontinued treatment because of adverse events. Six patients died during follow-up, 3 of them (UPN 1, 17 and 21) because of a solid tumor (glioblastoma, colon and thyroid cancer) that occurred 26, 19 and 14 months after the last cycle of Chl-R. The other three patients (UPN 3, 13 and 15) died because of Richter transformation, progressive disease, and myocardial infarction.

## Discussion

We report an OR rate of 74% (CR rate of 26%) among 27 elderly/unfit B-CLL pts treated front-line with Chl-R. The median age of the patients in our series was 72 years. The treatment schedule was well tolerated, allowing us to complete the treatment plan in 24 of 27 patients (89%). We recorded grade 3–4 neutropenia in 18.5% of the patients and only mild extra-hematological toxicities, including nausea in 7.4%, infusion related reaction in 15%, and vomiting in 3.7% of the patients. Only one patient developed fever of unknown origin that was resolved with treatment on an outpatient basis. No patients required reduction of dose or delay of therapy.

The standard of care for young and fit patients with B-CLL is the FCR regimen. In the GCLLSG CLL8 trial, Hallek et al showed that FCR is superior to FC, resulting in an ORR of 90% and a 3-year PFS of 51.8 months compared to an ORR of 80% and a 3-year PFS of 32.8 months for FC (p<0.0001).^4^ However, a high rate of adverse events were also reported in this study, including a 56% rate of grade 3–4 hematological toxicity and a 25% rate of infection, with a rate of adverse events higher in pts older than 65 years.^4^

Likewise, Tam et al showed that age older than 70 years was a factor adversely affecting the CR rate (p=0.02) and that patients 70 years or older were significantly less likely to complete 6 cycles of therapy (46% vs. 79% for patients < 70 years, *p=0*.001), with early cessation of treatment due to prolonged cytopenia. Tam et al also showed a high risk of late infectious complications following the FCR regimen.^6^

Because of the significant toxicity of the FCR regimen, monotherapy with Chlorambucil or Rituximab is widely used as first-line treatment in elderly or unfit patients with CLL. Both drugs are well tolerated, but overall response rates are relatively modest and complete responses are rare. For example, in the study of Hainsworth et al, Rituximab monotherapy resulted in a 51% ORR with only 4% CRs.^14^ Data reported in the literature on Chl monotherapy describe a higher OR rate (70%), but again with very few CRs (7%).^7^ For this reason, development of more effective treatment options for this patient population is required. The results obtained in our study suggest that the Chl-R combination could be one such option, especially considering that the considerably greater activity of this regimen was not associated with increased toxicity compared to treatment with each agent alone.

The findings of our study are similar to preliminary data of two ongoing multicenter trials with Chl and RTX in elderly patients with CLL that were presented at the American Society of Hematology 2010 scientific meeting.^15^,^16^ In the study of Hilmen et al, which included 100 pts with similar characteristics as those in our study (median age 70 years, range 43–86 vs median age 72 years, range 58 to 85, respectively), the ORR was 82%, with 9% achieving a CR, 58% achieving a PR, and 15% achieving a nodular PR.^15^ This ORR was 16% higher than the ORR of the matched subset of Chl-treated pts from the UK LRF CLL4 trial, further suggesting that the addition of Rituximab results in improved responses compared with Chl-alone. Grade 3–4 neutropenia was more frequent in the study of Hillmen et al than in our study (39% vs 18.5%, respectively), presumably because of the higher dose of Chl that was used (70mg/m^2^/cycle vs 1mg/kg/cycle, respectively). In the study of the Italian cooperative group, which included 85 elderly (>65 years) or 60–65 years old fludarabine-ineligible patients with CLL, the ORR was 81.2% with 16.5% of the patients achieving a CR.^16^

Recently, the German CLL study group published the results of a phase 2 trial investigating the safety and efficacy of bendamustine and rituximab (BR) in previously untreated patients with CLL.^17^ A total of 117 patients were enrolled, with a median age of 64 years (range, 34–78). The OR rate (88%) was slightly higher than in our study, with a similar rate of CRs (23.1%). The occurence of grade 3–4 neutropenia (19.7%) was similar to our study, but grade 3–4 thrombocytopenia (22.2%), anemia (19.7%) and infections (7.7%) were more frequent. Although the median age of the patients in the German CLL study was lower than in our series, the presented data suggest that B-R could be another effective and safe option for patients unfit for treatment with Fludarabine based regimens.

In conclusion, our study shows that the Chl-R combination is a safe and effective therapeutic option for untreated B-CLL pts that are not eligible for fludarabine treatment because of age and/or comorbidities. Further studies evaluating Chl-R and BR are required to determine which is the most appropriate currently available treatment for this large subset of patients with CLL.

## Figures and Tables

**Table 1:** Patients characteristics

UPN	SEX	AGE	STAGE	PBL	IgVH	BULKY	CD38	ZAP-70	FISH
**1**	M	68	C/IV	60890	MUT	-	NEG	NEG	NEG
**2**	F	64	A/I	93000	MUT	-	NEG	NEG	Del(13)
**3**	F	63	B/II	15680	-		NEG	NEG	NEG
**4**	M	80	B/II	51100	MUT	POS	NEG	NEG	del(13)
**5**	M	60	B/II	96000	UNM	-	POS	POS	+12
**6**	F	65	C/IV	18270	UNM	-	POS	NEG	del(13)
**7**	M	74	C/IV	6070	-	-	NEG	NEG	NEG
**8**	F	66	B/II	81840	MUT	POS	NEG	NEG	del(11)/+12
**9**	F	81	C/IV	4620	UNM	POS	POS	NEG	NEG
**10**	M	74	C/IV	19890	MUT	-	NEG	NEG	del(13)
**11**	F	68	C/IV	43950	UNM	-	POS	POS	del(13)
**12**	M	62	B/II	180000	MUT	-	NEG	NEG	del(13)
**13**	M	81	C/IV	9410	-	-	POS	NEG	NEG
**14**	M	81	B/II	50000	UNM	-	NEG	POS	NEG
**15**	M	76	B/II	76260	MUT	-	NEG	NEG	NEG
**16**	M	67	A/I	92000	UNM	-	POS	NEG	del(13)
**17**	F	72	A/I	102000	-	-	NEG	NEG	del(13)
**18**	F	73	A/I	45430	UNM	-	NEG	NEG	del(13)
**19**	M	75	B/I	17952	UNM	POS	NEG	NEG	del(11)/(13)
**20**	M	58	B/II	45120	UNM	-	NEG	POS	+12/del(13)
**21**	M	59	B/II	126000	UNM	-	NEG	POS	del(13)
**22**	M	79	C/IV	18020	UNM	-	POS	POS	del(11)
**23**	M	70	C/IV	6720	-	POS	NEG	-	del(13)
**24**	M	60	A/I	118000	UNM	-	NEG	NEG	del(13)
**25**	M	72	B/II	101556	MUT	-	NEG	NEG	del(13)
**26**	M	78	B/II	82600	UNM	-	POS	POS	del(13)
**27**	F	85	A/I	49220	UNM	-	POS	POS	NEG

UPN: unique patient number; M: male; F: female; PBL: peripheral blood lymphocyte (n/mmc); mut: mutated; unmut: unmutated; FISH: fluorescence in situ hybridization; +12: trisomy 12; Neg: normal karyotype; Del(13): deletion 13q14; Del(11): deletion 11q; -: not available.

**Table 2:** Results

	ORR	p values	PFS	p value	TTR	p value	OS	p value

**AGE (n= 27)**								
**< 70 (n=13)**	76.9		nr		nr		nr	
**> 70 (n=14)**	60.0	0.228	23	**0.099**	24	0.236		0.996

**CIRS (n=27)**								
**1–6 (n=18)**	77.8		32		34		nr	
**≥7 (n=9)**	66.7	0.535	35	0.185	25	0.175		0.328

**FISH (n= 27)**								
**STANDARD-RISK (n=24)**	75.0		32		34		nr	
**HIGH-RISK (n=3)**	66.7	0.756	18	**0.064**	24	0.169		0.381

**IGVH (n=22)**								
**MUT (n=8)**	75.0		32		34		nr	
**UNM (n=14)**	78.6	0.848	nr	0.714	24	0.973		0.485

**BULKY (n=27)**								
**NO (n=22)**	72.7		32		34		nr	
**YES (n=5)**	80.0	0.738	21	0.115	24	0.155		0.796

**CD38 (n=27)**								
**POS (n=9)**	66.7		nr		nr		nr	
**NEG (n=18)**	77.8	0.535	25	0.280	32	0.485		0.300

**Zap-70 (n=26)**								
**POS (n=8)**	75.0		25		34		nr	
**NEG (n=18)**	72.2	0.883	29	0.636	32	0.633		0.708

ORR: Overall Response Rate; PFS: Progression Free Survival; TTT: Time To Retreatment; OS: Overall Survival; nr: not reached.

**Table 3:** Treatment-related toxicity

ADVERSE EVENTS	TOTAL NUMBER OF PATIENTS (%)[Grade 3–4]	TOTAL NUMBER OF PATIENTS (%)[Grade 1–2]

**Hematological**		
**Neutropenia**	5 (18.5%)	n.e.
**Thrombocytopenia**	-	n.e.
**Autoimmune hemolityc anemia**	1 (3.7%)	n.e.
**Autoimmune hemolityc thrombocytopenia**	-	n.e.
**Anemia**		

**Extra-hematological**		
**Nausea**	-	2 (7.4%)
**Vomiting**	-	1 (3.7%)
**Infusion related reaction**	-	4 (15%)
**Infections, total**	-	2 (7.4%)

n.e.: not evaluated
